# Identification of a cytogenetic and molecular subgroup of acute myeloid leukemias showing sensitivity to L-Asparaginase

**DOI:** 10.18632/oncotarget.18565

**Published:** 2017-06-19

**Authors:** Salvatore Nicola Bertuccio, Salvatore Serravalle, Annalisa Astolfi, Annalisa Lonetti, Valentina Indio, Anna Leszl, Andrea Pession, Fraia Melchionda

**Affiliations:** ^1^ Pediatric Hematology and Oncology Unit, Department of Pediatrics, S.Orsola-Malpighi Hospital, University of Bologna, Bologna, Italy; ^2^ “Giorgio Prodi” Cancer Research Center, University of Bologna, Bologna, Italy; ^3^ Department of Woman and Child Health, Laboratory of Hematology-Oncology, University of Padova, Padova, Italy

**Keywords:** acute myeloid leukemia, monosomy chromosome 7, L-Asparaginase, ASNS gene

## Abstract

L-Asparaginase (L-Asp) is an enzyme that catalyzes the hydrolysis of L-asparagine to L-aspartic acid, and its depletion induces leukemic cell death. L-Asp is an important component of treatment regimens for Acute Lymphoblastic Leukemia (ALL). Sensitivity to L-Asp is due to the absence of L-Asparagine synthetase (ASNS), the enzyme that catalyzes the biosynthesis of L-asparagine. *ASNS* gene is located on 7q21.3, and its increased expression in ALLs correlates with L-Asp resistance. Chromosome 7 monosomy (-7) is a recurrent aberration in myeloid disorders, particularly in adverse-risk Acute Myeloid Leukemias (AMLs) and therapy-related myeloid neoplasms (t-MN), that leads to a significant downregulation of the deleted genes, including *ASNS*. Therefore, we hypothesized that -7 could affect L-Asp sensitivity in AMLs. By treating AML cell lines and primary cells from pediatric patients with L-Asp, we showed that -7 cells were more sensitive than AML cells without -7. Importantly, both ASNS gene and protein expression were significantly lower in -7 AML cell lines, suggesting that haploinsufficiency of *ASNS* might induce sensitivity to L-Asp in AMLs. To prove the role of ASNS haploinsufficiency in sensitizing AML cells to L-Asp treatment, we performed siRNA-knockdown of *ASNS* in AML cell lines lacking -7, and observed that *ASNS* knockdown significantly increased L-Asp cytotoxicity. In conclusion, -7 AMLs showed high sensitivity to L-Asp treatment due to low expression of ASNS. Thus, L-Asp may be considered for treatment of AML pediatric patients carrying -7, in order to improve the outcome of adverse-risk AMLs and t-MN patients.

## INTRODUCTION

Cytogenetic abnormalities represent the strongest independent predictors for response to therapy and survival in patients with Acute Myeloid Leukemia (AML), as a complex aberrant karyotype is associated with poor prognosis [1]. Adverse-risk cytogenetic abnormalities occur in 20–30% of *de novo* AML and 70% of therapy-related myeloid neoplasm (t-MN) [2–4]. Outcomes in pediatric AML has increased remarkably over the past two decades due to intensive chemotherapy, improved supportive therapy and a better risk stratification [4–7]. Despite these important progresses, overall survival is below 75% with a relapse rate up to 40% [7–8]. The most common high-risk cytogenetic abnormality is monosomy of chromosome 7 (-7), identified in half of adverse-risk *de novo* AML and t-MN patients [4]. It was shown that this event leads to a significant down regulation of the genes located in the deleted region, including tumor suppressors *CUX1* [6], *MLL3* [9–10] and *L-Asparagine synthetase* (*ASNS*) [11].

The gene coding for *ASNS* is located on 7q21.3 and catalyzes the biosynthesis of L-asparagine from L-aspartate in an ATP-dependent reaction for which L-glutamine is the nitrogen source under physiological conditions [12]. Neoplastic cells require large amount of asparagine to maintain their malignant growth, and they obtain this amino acid both from blood serum and through *de novo* synthesis of asparagine via ASNS. Even if the regulation of amino acid uptake is not completely understood, recent findings indicate that asparagine may play a role in cellular amino acid homeostasis, and this is why intracellular asparagine levels are critical for cancer cell growth [13].

However, it has been observed that in some cases neoplastic cells lack or have very low levels of the enzyme ASNS and are not able to synthesize asparagine *de novo*; hence, for proliferation and survival, they are exclusively dependent on extracellular asparagine supply. For example, primary Acute Lymphoblastic Leukemia (ALL) cells and many ALL cell lines exhibit a particularly low level of *ASNS* expression and thus are unusually sensitive to asparagine depletion.

L-Asparaginase (L-Asp) is an enzyme that catalyzes the hydrolysis of L-asparagine to L-aspartic acid and ammonia, resulting in depletion of asparagine and ultimately in tumor cells death. Clinical data published over the last two decades suggested L-Asp is a very important drug for ALL treatment, and indeed L-Asp is part of standard therapeutic protocols for remission induction and intensification in pediatric ALL regimens and in the majority of adult ALL treatment protocols [14]. Sensitivity to L-Asp is due to the absence of ASNS enzyme in ALL cells. Several studies focused on a possible correlation between L-Asp sensitivity and the DNA methylation status of the *ASNS* gene, demonstrating that 74% of B and 83% of T cells displayed methylation of the *ASNS* promoter [15]. On the other hand, increased expression of *ASNS* has been correlated with *in vitro* resistance to L-Asp in ALL cells [12].

The rationale of this study is based on the hypothesis that the cytogenetic status of chromosome 7 in AML could affect the expression of *ASNS* and could sensitize AML cells to L-Asp treatment. From this perspective, -7 could be used as a molecular marker to select AML patients eligible for L-Asp treatment in order to improve the outcome of adverse-risk AML and t-MN pediatric patients.

## RESULTS

### FISH analysis identifies AML cell lines with monosomy of chromosome 7

**Figure 1:**
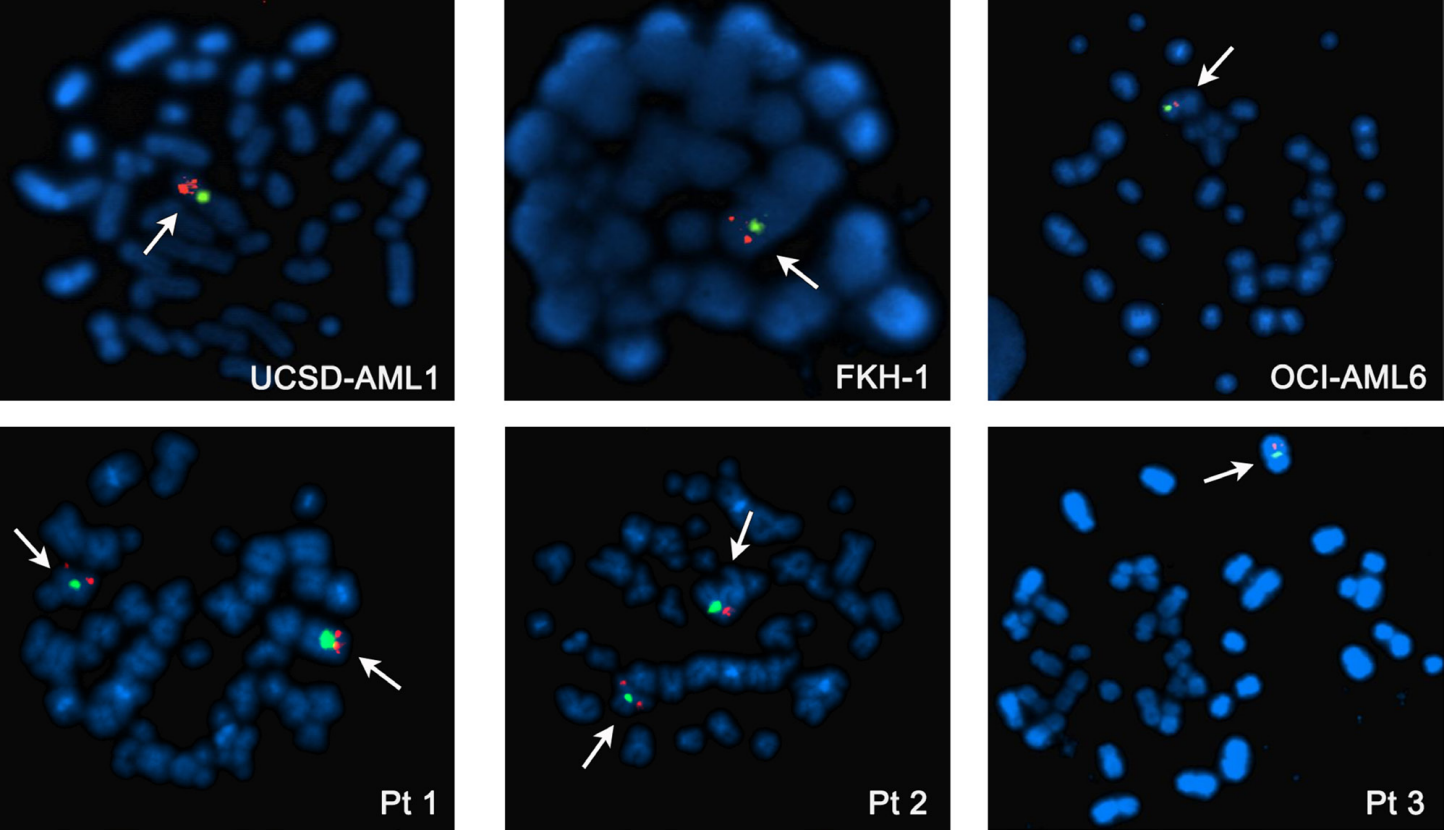
Metaphase FISH analysis of AML cell lines with -7 (upper panel) and AML patients (lower panel) Normal diploid chromosome 7 has two signals for both centromeric (green) and telomeric (orange) probes. Patients 1 and 2 are disomic, whereas Patient 3, UCSD-AML1, FKH-1 and OCI-AML6 cell lines have -7.

FISH analysis was performed to evaluate the status of chromosome 7 in 11 AML cell lines, 7 ALL cell lines, and primary cells derived from 3 AML pediatric patients. Analysis of interphase and metaphase identified -7 in 3/11 AML cell lines (UCSD-AML1, FKH-1, OCI-AML6) and 1/3 AML primary samples (Figure 1). 2/11 AML cell lines were disomic (KASUMI-1 and MOLM-13), whereas the remaining AML cells had aberrations involving chromosome 7 and including trisomies, duplications and translocations (Supplementary Figure 1). Of note NOMO-1 cells, carrying a translocation of chromosome 7 that causes the loss of the distal arm of 7q, are nonetheless disomic at the *ASNS* locus, as shown by qPCR (Supplementary Figure 1D). None of ALL cell lines had -7, but tetrasomy of chromosome 7 was identified in 5/7 ALL cells (DND-41, MOLT-4, RPMI-8402, CCRF-CEM, JURKAT) (Supplementary Figure 1).

### Monosomy of chromosome 7 affects ASNS expression and correlates with high sensitivity to L-Asp

**Figure 2:**
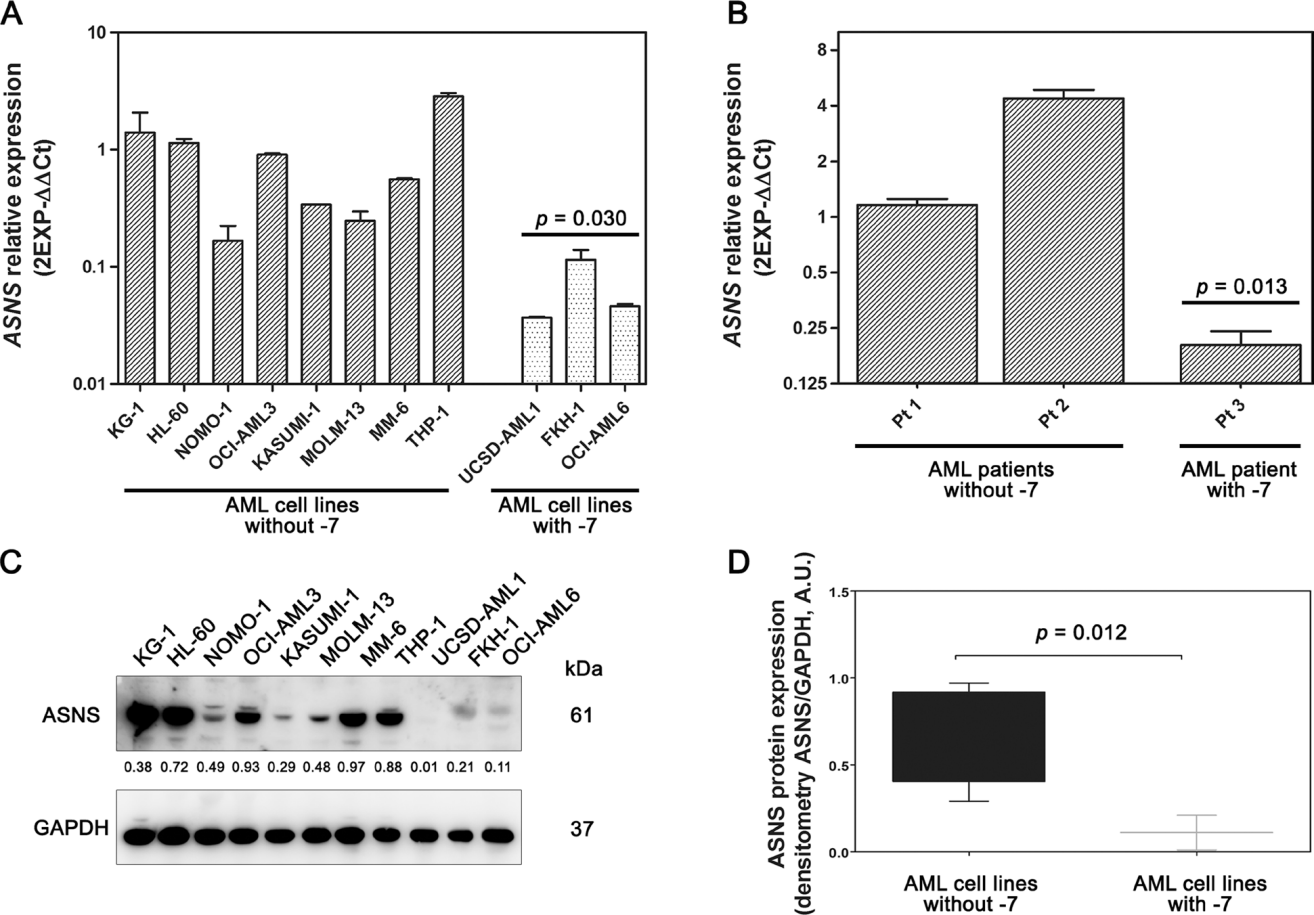
Gene and protein expression of ASNS in AML samples AML cell lines (**A**) and AML primary cells (**B**) with -7 displayed low amount of *ASNS* transcript. Bars represent 2^−ΔΔCt^ value. Gene expression was normalized to *GAPDH* and *ATP5B* housekeeping genes. Data are expressed as mean ± SD of three independent experiments. Statistical analysis (Unpaired *t* test) was performed to compare mean relative mRNA abundance between AML cells, with or without -7. (**C**) Western blot analysis showing low amount of ASNS protein in AML cell lines with -7. Thirty micrograms of protein were blotted to each lane. Antibody to GAPDH served as a loading control. (**D**) The amount of ASNS protein, determined by densitometric analysis and normalized to GAPDH density, was significantly lower in AML cells with -7 (*p* = 0.012, Mann Whitney test).

**Figure 3:**
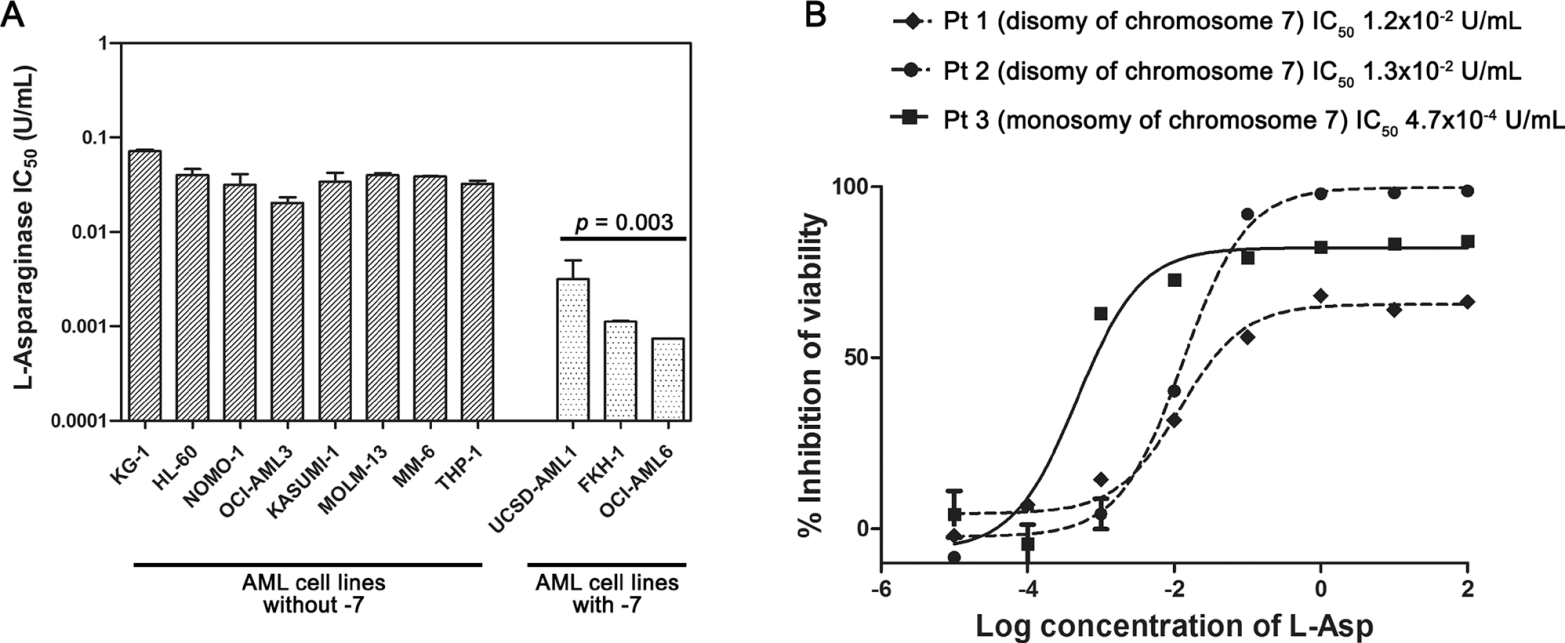
L-Asp sensitivity of AML cell lines and primary AML cells with or without -7 (**A**) AML cell lines with -7 (UCSD-AML1, FKH-1 and OCI-AML6) were more sensitive to L-Asp than AML cell lines without -7 (*p* = 0.003). (**B**) Dose-response growth curves of primary AML cells treated with L-Asp showed that AML -7 are more sensitive than other AML primary cells. Pt1: AML M4 with disomy of chromosome 7; Pt2: AML M2 with disomy of chromosome 7; Pt3: AML M2 with monosomy of chromosome 7.

To investigate the effects of -7, we analyzed gene and protein expression of ASNS in both AML cell lines and primary samples. *ASNS* gene expression was evaluated by qRT-PCR. AML cell lines and primary samples with -7 had a significantly lower amount of *ASNS* transcript compared to those without such cytogenetic abnormality (*p* = 0.030 and *p* = 0.013, respectively) (Figure 2A and 2B). *ASNS* expression was significantly downregulated also in the dataset from the TARGET AML database including about 250 samples from pediatric AML (Supplementary Figure 2). Importantly, -7 also resulted in a drastic reduction of ASNS protein (*p* = 0.012) (Figure 2C and 2D). To assess whether AML cells with -7 could be sensitive to L-Asp treatment, these were cultured with increasing concentrations of L-Asp ranging from 0.0001 U/mL to 100 U/mL and cell viability was evaluated after 48 hours treatment. IC_50_ values ranged between 10^–2^ and 10^–3^ U/mL. In particular, AML cells with -7 were more sensitive to L-Asp treatment than AML cells without -7 (*p* = 0.003) (Figure 3A). Similarly, we treated for 72 hours AML primary cells with L-Asp, and detected a lower IC_50_ in Pt 3 with monosomy of chromosome 7 (10^–4^ U/mL), in comparison to Pts 1 and 2 (10^–2^ U/mL) disomic for chromosome 7 (Figure 3B). Of note, IC_50_ values of AML cell lines and AML patient sample with -7 were quite different (10^–3^ U/mL and 10^–4^ U/mL, respectively). However, this result is in accordance with a recent study which reported that ALL patient samples were significantly more sensitive to L-Asp compared to cell lines [16]. Indeed, despite the basis for differences in L-Asp sensitivity remains unclear, several genomic interindividual variations in specific pathways, including aspartate metabolism pathway, can contribute to L-Asp sensitivity [16]. Because *ASNS* gene expression correlated with both ASNS protein expression and sensitivity to L-Asp (Spearman *r* = 0.709 and *r* = 0.691, with *p* = 0.015 and *p* = 0.019, respectively), overall these data suggested that L-Asp sensitivity is related to ASNS haploinsufficiency.

### In AML cells ASNS haploinsufficiency is caused exclusively by chromosome 7 deletion

**Figure 4:**
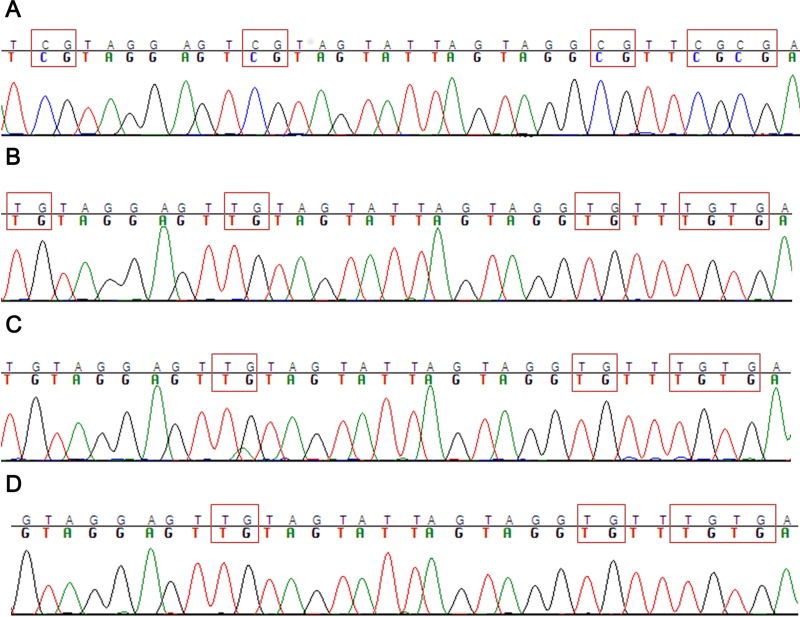
Methylation analysis of *ASNS* CpG (**A**) Example of ALL cell lines with ASNS promoter hypermethylated (DND-41). (**B**) Example of ALL cell line with ASNS promoter hypomethylated (CCRF-CEM). (**C**) Example of AML cell line with -7 and with *ASNS* promoter hypomethylated (UCSD-AML1). (**D**) Example of other AML cell line without -7 and with *ASNS* promoter hypomethylated (MOLM-13).

**Figure 5:**
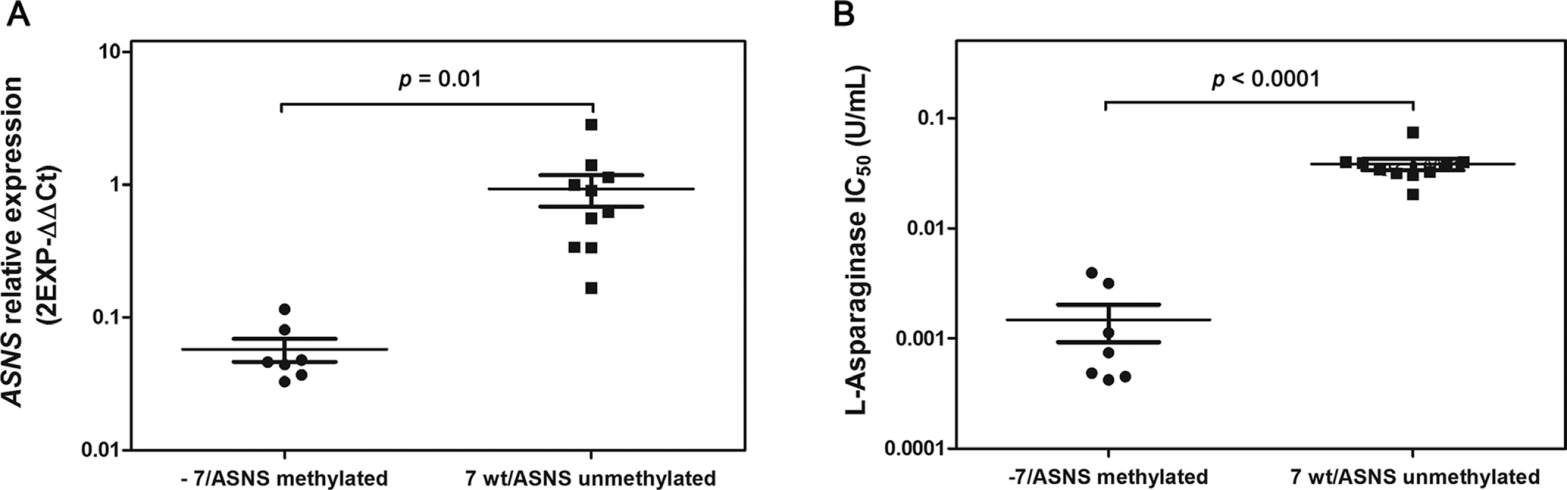
(**A**) Low expression of *ASNS* gene correlated with *ASNS* promoter methylation (in ALL cells) or -7 (in AML cells). Cell lines with -7 or methylation of *ASNS* promoter (●) have lower *ASNS* expression than cell lines without these genetic aberrations (■). (**B**) Cell lines with -7 or methylation of *ASNS* promoter (●) displayed higher sensitivity to L-Asp treatment than other cell lines without these genetic aberrations (■).

There are alternative mechanisms that can contribute to *ASNS* gene silencing. For example, different studies demonstrated that in ALL *ASNS* promoter methylation correlated with lack of *ASNS* expression, and consequently *ASNS* methylation may underlie the susceptibility of ALL cells to L-Asp chemotherapy [15, 17]. Therefore, we analyzed the methylation level of *ASNS* gene promoter in ALL cell lines and AML cells with -7. Some ALL cell lines (DND41, HPB-ALL, MOLT4, RPMI-8402) showed complete methylation of the *ASNS* promoter, as opposed to other ALL cells (SEM, JURKAT, CCRF-CEM) (Figure 4A and 4B). Importantly, ALL cell lines exhibiting promoter methylation were characterized by higher sensitivity to L-Asp and lower expression of *ASNS* transcript (Supplementary Figure 3A and 3B). In contrast, none of the AML cell lines showed promoter methylation (Figure 4C and 4D), further supporting the hypothesis that -7 is the sole event responsible for *ASNS* downregulation and haploinsufficiency in AML cells. Overall, these results indicated that low expression of *ASNS* is associated to -7 and hypermethylation of *ASNS* promoter in AML and in ALL cells, respectively, and that low expression of *ASNS* is related to high sensitivity to L-Asp (Figure 5A and 5B).

### ASNS downregulation leads to L-Asp sensitization of AML cells without chromosome 7 deletion

**Figure 6:**
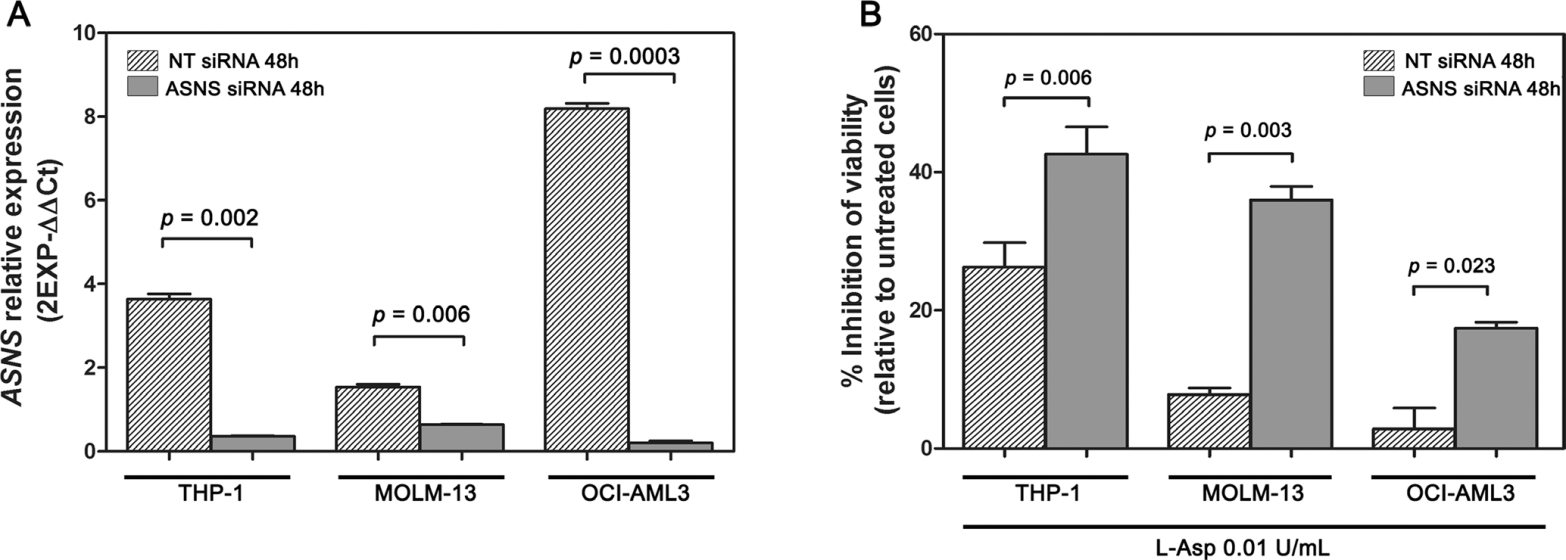
Knockdown of *ASNS* gene with specific siRNA followed by L-Asp treatment (**A**) Expression of *ASNS* gene after 48 hours from siRNA transfection. (**B**) Inhibition of cell viability following 48 hours treatment with 0.01 U/mL of L-Asp. AML cell lines transfected with *ASNS* siRNA showed a higher mortality than AML cell lines transfected with NT siRNA.

In order to ascertain whether in AML cells monoallelic loss of *ASNS* is the main reason for cytotoxicity induced by L-Asp, we transiently silenced *ASNS* gene in THP-1, MOLM-13 and OCI-AML3 cell lines using RNA interference techniques. AML cells transfected with a non-targeting (NT) control siRNA (NT siRNA) were used as control. Significant suppression (> 50%) of *ASNS* expression after transfection was confirmed by RealTime PCR (Figure 6A). Following transfection, the cells were treated with 0.01 U/mL of L-Asp for 48 hours. AML cell lines transfected with *ASNS* siRNA showed a significant inhibition of cell viability compared to those transfected with NT siRNA (*p <* 0.01) (Figure 6B). These data indicated that *ASNS* is a critical mediator of cytotoxicity induced by L-Asp in AML cells with -7.

## DISCUSSION

Monosomy of chromosome 7 is a rare but well-described cytogenetic abnormality associated with poor outcome in pediatric AML [1], therefore allogeneic hematopoietic stem cell transplantation may be recommended for patients in first hematological remission [18].

L-Asp is an important chemotherapeutic agent for management of ALL, but it is not part of chemotherapy of AML. Normal cells are protected from asparagine requirement due to their ability to produce this amino acid [19]. The enzyme that catalyzes the biosynthesis of L-asparagine is ASNS, located on 7q21.3. Importantly, low expression of *ASNS* transcript has been described in B and T-lineage ALL, and correlates to their high sensitivity to L-Asp treatment [20–21]. Accordingly, resistance to L-Asp therapy may be linked to high level of *ASNS* gene expression, that allows the production of asparagine. Increased gene expression of *ASNS* can be stimulated by the tumor cells after exposure to L-Asp, otherwise bone marrow mesenchymal stromal cells, that represent the leukemic cells microenvironment, can express high levels of ASNS [20, 22]. Indeed, co-culture with mesenchymal cells protected ALL cells from L-Asp cytotoxicity [23]. There are only few studies testing *in vitro* efficacy of L-Asp in AML, showing sensitivity to L-Asp in some FAB subgroups, including M1 (Acute myeloblastic leukemia with minimal maturation), M4 (Acute myelomonocytic leukemia) and in particular in the M5 subgroup (Acute monocytic leukemia), that showed low expression of *ASNS* [24–26]. In this scenario our work represents the first preclinical study testing sensitivity to L-Asp in an AML subgroup carrying a specific molecular marker, monosomy of chromosome 7, that strongly correlates with poor prognosis. Etiology of AML with -7 was linked to haploinsufficiency of genes mapped on chromosome 7 including *ASNS* [11]. So we hypothesized that -7 AML could show a higher sensitivity to L-Asp treatment.

We treated AML cell lines and AML primary samples, with or without -7, with L-Asp and observed a significantly higher sensitivity in cell lines and primary cells with -7. The sensitivity of AML cell lines and primary cells correlated with expression of ASNS. In particular AML with -7 had a lower expression of ASNS than other types of AML. Therefore our results support the existence of a positive correlation between low ASNS expression and high sensitivity to L-Asp in AML. There are different mechanisms that can be involved in down regulation of *ASNS* gene. For example, in ALL, *ASNS* promoter methylation induces low *ASNS* expression, resulting in susceptibility of ALL cells to L-Asp chemotherapy [15, 17]. We showed that some ALL cell lines (DND41, HPB-ALL, MOLT4 and RPMI8402) had hypermethylated *ASNS* promoter. These ALL cell lines had lower expression of *ASNS* gene, and exhibited higher L-Asp sensitivity. In contrast none of the AML cell lines showed promoter methylation, further supporting the finding that *ASNS* inhibition is only due to -7. To confirm the correlation between low *ASNS* expression and high sensitivity to L-Asp in AML with -7, we inhibited *ASNS* expression using a specific siRNA before L-Asp treatment in three different AML cell lines. In all cases, transfection with specific siRNA for *ASNS* gene increased cell sensitivity to L-Asp treatment compared to mock-transfected cells.

In conclusion these results may indicate that AML with -7 have high sensitivity to L-Asp treatment due to low expression of ASNS. Low expression of *ASNS* in AML cell lines is linked to -7 and haploinsufficiency of *ASNS* gene. Although other studies are necessary to understand if other genes mapping on chromosome 7 may contribute to L-Asp sensitivity, L-Asp could be evaluated as a novel opportunity for therapeutic intervention in pediatric AML with -7 that are characterized by a poor outcome and low sensitivity to conventional chemotherapy.

## MATERIALS AND METHODS

### Cell culture and reagents

Human ALL cell lines (DND41, HPB-ALL, MOLT-4, RPMI-8402, CCRF-CEM, JURKAT) and AML cell lines (KG-1, HL-60, NOMO-1, KASUMI-1, MOLM-13, THP-1, FKH-1) were grown in RPMI1640 (Lonza) supplemented with 10% Fetal Bovine Serum (Invitrogen), *2 mM L-glutammine,*100 U/mL penicillin and 100 μg/mL streptomycin (GIBCO), SEM cell line was grown in Iscove medium (Sigma) supplemented with 20% FBS (Invitrogen). OCI-AML3 and OCI-AML6 were grown in Alpha-MEM (Lonza). UCSD-AML1 was supplemented with GM-CSF 10 ng/ml (Sigma) and MM-6 (Mono-Mac-6) was supplemented with OPI (Sigma). Primary cells derived from AML patients were cultured with Chang Marrow medium (Technogenetics). All cell lines were grown at 37°C in a humidified atmosphere of 5% CO2. L-Asp (Erwinase) was purchased by Jazz Pharmaceutical. The patients analyzed were enrolled in the Associazione Italiana Ematologia Oncologia Pediatrica (AIEOP) 2002/01 AML Study [2] after obtaining written informed consent from the parents according to the Declaration of Helsinki. Pt1 was classified as AML FAB M4 (Acute myelomonocytic leukemia) with chromosome 7 disomy; Pt 2 was a AML FAB M2 (Acute myeloblastic leukemia with maturation) with disomy of chromosome 7; Pt 3 was a AML FAB M2 with monosomy of chromosome 7.

### FISH analysis

FISH experiments were performed on all cell lines following the Vysis specific probe protocol (Vysis, Downers Groove, USA) and using the LSI D7S522/CEP 7 probe, that contains a mixture of a Spectrum Orange D7S522 probe (7q31) and a Spectrum Green CEP 7 probe (7p11.1-q11.1).

### RNA extraction and quantitative PCR

Total RNA was extracted by RNeasy spin column method (Qiagen). 1 μg total RNA was reverse transcribed to single-stranded cDNA using the Transcriptor first strand cDNA synthesis kit (Roche Diagnostics) with oligo-dT primers (2.5 μM). Gene-specific primers amplifying *ASNS* were designed with Primer Express 3.0 Software (Applied Biosystems, Monza, Italy) and qRT-PCR was performed using FastStart Sybr Green (Roche Diagnostics) on the Light Cycler 480 apparatus (Roche Diagnostics). ΔΔCt method was used to quantify gene expression levels relative to two housekeeping genes, *GAPDH* and *ATP5B*. Quantitative RT-PCR primer sequences were as follows: *ASNS* forward (5′→3′) GACAGAAGGATTGGCTGCCT, *ASNS* reverse (3′→5′) CATCCAGAGCCTGAATGCCT, *GAPDH* forward (5′→3′) CCAATATGATTCCACCC ATGGC, *GAPDH* reverse (3′→5′) CTTGATTTTC GAGGGATCTCGC, *ATP5B* forward 5′→3′) 5′ GTCTT CACAGGTCATATGGGGA-3′, *ATP5B* reverse (3′→5′)-ATGGTCCCACCATATAGAAGG -3′.

### Western blotting

Cells were lysed in M-PER™ Mammalian Protein Extraction Reagent (Thermo Fisher Scientific Inc., Rockford, IL, USA) supplemented with the Protease and Phosphatase Inhibitor Cocktail (Thermo Fisher Scientific Inc., Rockford, IL, USA). For Western blot analysis, equal amounts of protein (30 μg) were separated by SDS-PAGE and then electro-transferred to nitrocellulose membrane. The membranes were blocked with 5% milk at room temperature for 1 hour and incubated over night at 4°C with primary antibodies directed towards GAPDH (Cell Signaling Techonology) and ASNS (Thermo Fischer Scientific). Anti-rabbit HRP-conjugated secondary antibody was from Cell Signaling Techonology. Protein bands were visualized using the ECL Prime Western Blotting Detection Reagent (Amersham, IL, USA) and densitometry scanning of the bands was performed using a Chemidoc 810 Imager with the appropriate software (UVP, Upland, CA, USA).

### Cell viability assay

To test the effects of L-Asp, cell lines and primary AML cells were cultured for 48 or 72 hours, respectively, in the presence of increasing drug concentrations from 10^–5^ U/mL to 10^2^ U/mL. Assays were performed in triplicate and cell viability was determined using the WST1 (4-[3-(4-lodophenyl)-2-(4-nitrophenyl)-2H-5-tetrazolio]-1, 3-benzene disulfonate) cell proliferation kit (Roche Applied Science, Monza, Italy), according to manufacturer's instructions. Non-linear regression curve fit and IC_50_ analysis were calculated with Graphpad Prism software (San Diego, CA, USA).

### Methylation analysis

Genomic DNA was extracted using the QIAamp DNA kit (Qiagen). Methylation analysis was performed using the Epigentek Methylamp modification Kit (Epigentek). After bisulfite modification the *ASNS* promoter was amplified with specific primer: *ASNS* forward primer 5′ TTAGGGAATTAGGATAGAAAGG TTT 3′, *ASNS* reverse primer 5′ AAACAAACCAAATT CAAAAACCTCC 3′. PCR product was purified and labeled with BigDye Terminator 1.1 (Applied Biosystems) and sequenced on a ABI3730 instrument (Applied Biosystems).

### Copy number analysis by qPCR

ASNS copy number was estimated in NOMO-1 cell line compared to disomic (SEM, KASUMI-1, MOLM-13) and monosomic (UCSD-AML1, FKH-1) cell lines by qPCR with specific primer pairs on ASNS genomic DNA. ΔΔCt method was used to quantify copy number relative to PTMA promoter region as a reference locus.

### siRNA treatment

*ASNS* inhibition was performed by nucleofection on a Nucleofector II device (Lonza GmbH, Cologne, Germany) using 3 unique 27mer siRNA duplexes (Locus ID 440) (Origene). Mock transfection was performed with a non-targeting (NT) control siRNA (Origene). THP-1, MOLM-13 and OCI-AML3 cells were transfected with siRNAs at a final concentration of 200 nM. Following 3–5 hours from transfection, the cells were treated with 0.01 U/mL L-Asp for 48 hours and cell viability was determined using the WST1 cell proliferation kit (Roche Applied Science).

### Statistical analysis

Correlation between *ASNS* gene expression, ASNS protein expression and sensitivity to L-Asp were analyzed using Spearman rank test. To compare IC_50_, gene and protein expression values between different groups, we performed unpaired *t* test. All analyses were performed using Prism software version 6 (GraphPad Prism, San Diego, CA, USA). Statistical significance was considered at two-tailed *p <* 0.05.

## SUPPLEMENTARY MATERIALS FIGURES AND TABLES


