# Supplementary material for: Identification of a cytogenetic and molecular subgroup of acute myeloid leukemias showing sensitivity to L-Asparaginase
Source: Oncotarget. 2017 Jun 19;8(66):109915–23. doi: 10.18632/oncotarget.18565 (PMC5746353; doi:10.18632/oncotarget.18565)
Supplement: Supplementary file 1 [file oncotarget-08-109915-s001.pdf]

Identification of cytogenetic and molecular subgroup of acute myeloid leukemias showing sensitivity to L-Asparaginase

SUPPLEMENTARY MATERIALS

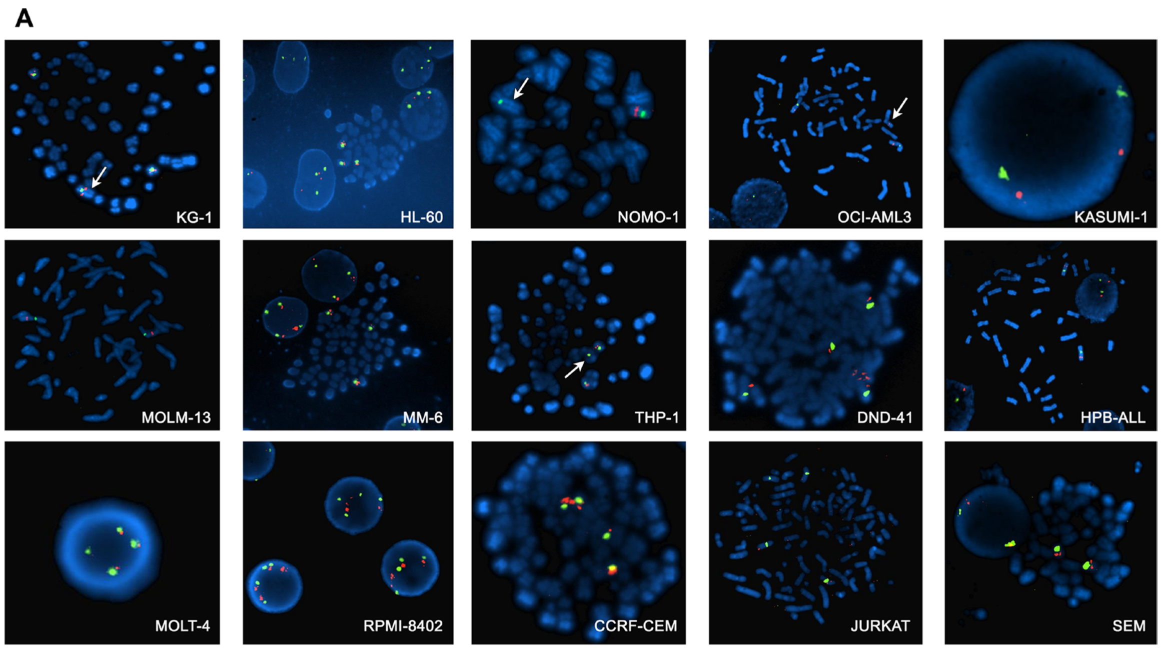

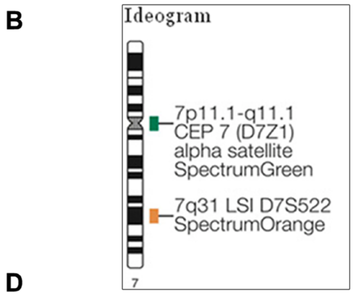

C

Cell line	Leukemia subtype	Cytogenetic status of chromosome 7
KG-1	AML	ish + der(7)t(7q;?)
HL-60	AML	trisomy
NOMO-1	AML	ish der(7)t(7q;?)
OCI-AML3	AML	ish dup(7)(qter->q31?) and translocation
KASUMI-1	AML	disomy
MOLM-13	AML	disomy
MM-6	AML	trisomy/tetrasomy
THP-1	AML	ish + 7/ish + der(7)t(7q;?) or i(7p)
DND-41	T-ALL	tetrasomy
HPB-ALL	T-ALL	disomy
MOLT-4	T-ALL	tetrasomy
RPMI-8402	T-ALL	tetrasomy
CCRF-CEM	T-ALL	tetrasomy
JURKAT	T-ALL	tetrasomy
SEM	B-ALL	disomy

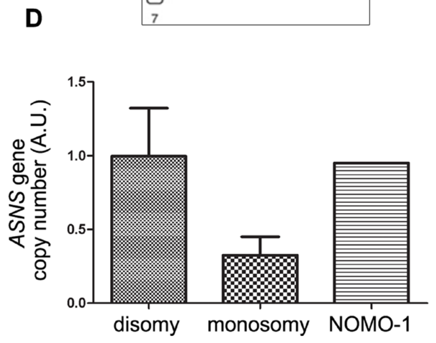

Supplementary Figure 1: FISH analysis of AML and ALL cell lines without -7, using LSI D7S522/CEP 7 probe, a mixture of Spectrum Orange (7q31) and Spectrum Green (7p11.1-q11.1) probes. Normal diploid chromosome 7 have two signals for both centromeric (green) and telomeric (orange) probes. (**A**) Interphase or metaphase FISH results of 8 AML (KG-1, HL-60, NOMO-1, OCI-AML3, KASUMI-1, MOLM-13, MM-6, THP-1) and 7 ALL (DND-41, HPB-ALL, MOLT-4, RPMI-8402, CCRF-CEM, JURKAT, SEM) cell lines. Arrows indicates complex aberrations involving chromosome 7. (**B**) Ideogram of LSI D7S522/CEP 7 probe. (**C**) Summary of cytogenetic status of chromosome 7 in both AML and ALL cell lines. (**D**) Copy number analysis by qPCR of ASNS locus in NOMO-1 cells, compared with disomic (SEM, KASUMI-1, MOLM-13) and monosomic (UCSD-AML1, FKH-1) cell lines.

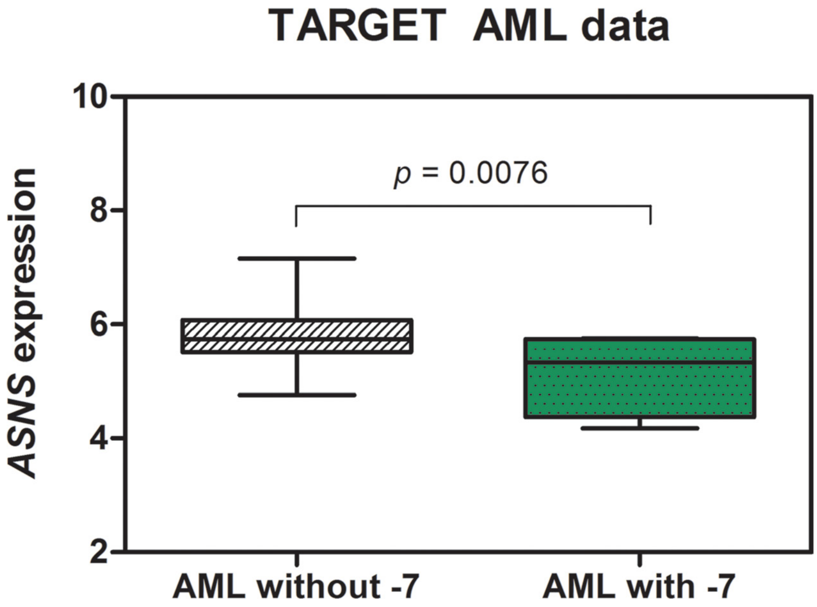

Supplementary Figure 2: ASNS expression in pediatric TARGET AML database. Boxplots show ASNS normalized expression of samples from the NCI-COG TARGET AML project (https://ocg.cancer.gov/programs/target/acute-myeloid-leukemia), classified by chromosome 7 status.

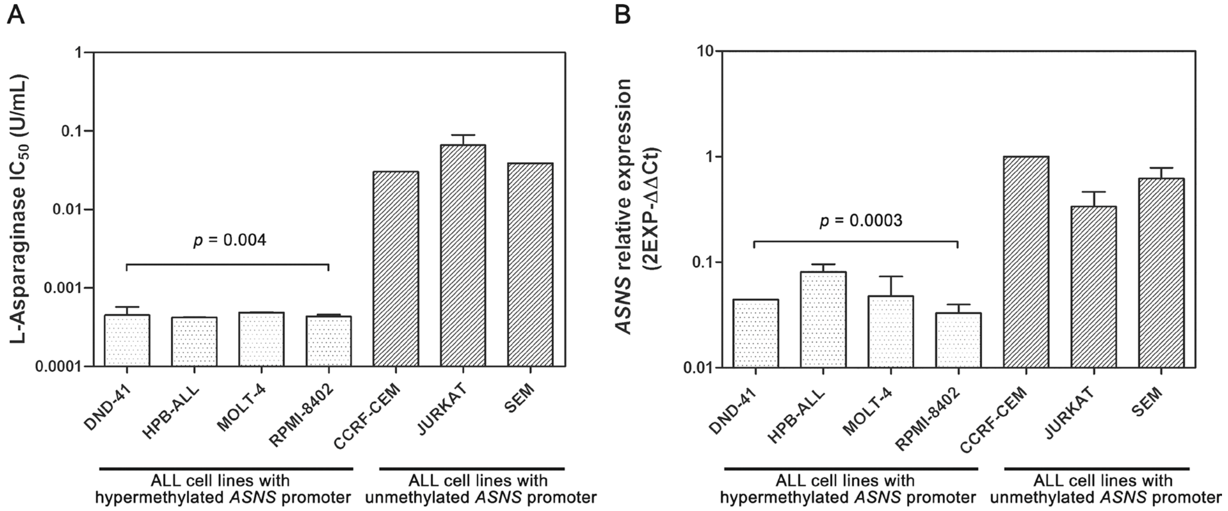

Supplementary Figure 3: (**A**) IC_{50} of ALL cell lines after 48 h of treatment with increasing concentrations of L-Asp. DND41, HPB-ALL, MOLT-4 and RPMI-8402, which have *ASNS* promoter methylation, were more sensitive than other ALL cell lines (Unpaired *t* test, $p = 0.004$). (**B**) ALL cell lines sensitive to L-Asp treatment showed lower amount of *ASNS* transcript (Unpaired *t* test, $p = 0.0004$). *ASNS* relative expression is calculated as $2^{-\Delta\Delta Ct}$. Gene expression was normalized to *GAPDH* and *ATP5B* housekeeping genes. Data are expressed as mean ± SD of three independent experiments.
